# Supplementary material for: Rational design of inducible CRISPR guide RNAs for de novo assembly of transcriptional programs
Source: Nat Commun. 2017 Mar 3;8:14633. doi: 10.1038/ncomms14633 (PMC5339017; doi:10.1038/ncomms14633)
Supplement: Supplementary Information — Supplementary Figures and Supplementary Tables [file ncomms14633-s1.pdf]

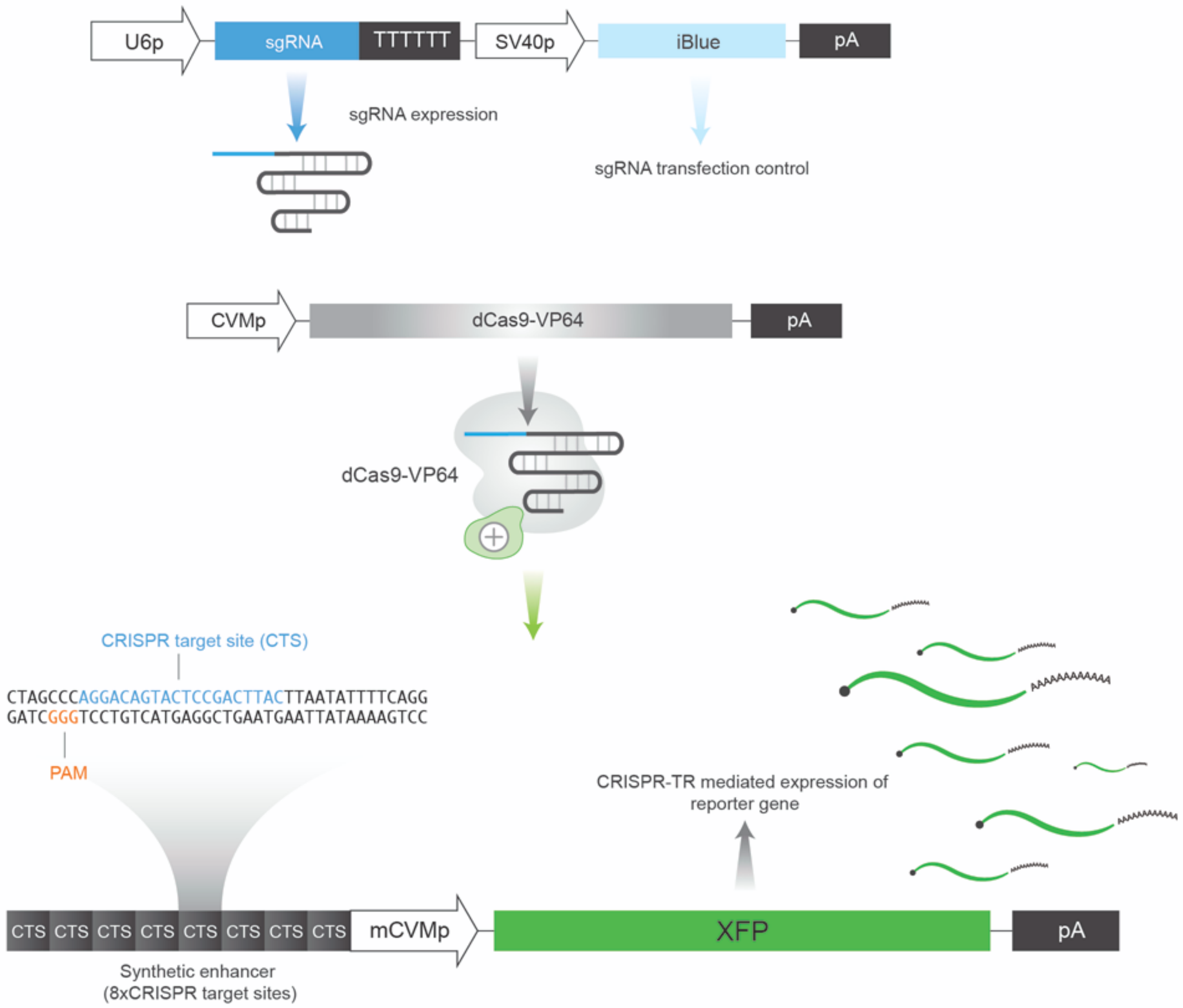


**Supplementary Figure 1. Schematic of CRISPR-TR reporter assay in HEK293-T cells.** A bicistronic vector is used to couple the expression of U6-driven sgRNAs with a fluorescent reporter (iBlue). The dCas9-VP64 gene is expressed under a CMV promoter from a separate plasmid. The assembled sgRNA-dCas9-VP64 complex is targeted to the synthetic enhancer (8 x CRISPR target sites (CTS)) driving the expression of a target gene (XFP), by programing the sgRNA spacer sequence. In this study two sgRNA spacers were programmed (CTS1, CTS2), to drive specific expression (with no detectable cross-talk) of two corresponding reporter target genes (8xCTS1-mCMVp-EYFP-pA and 8xCTS2-mCMVp-ECFP-pA respectively).

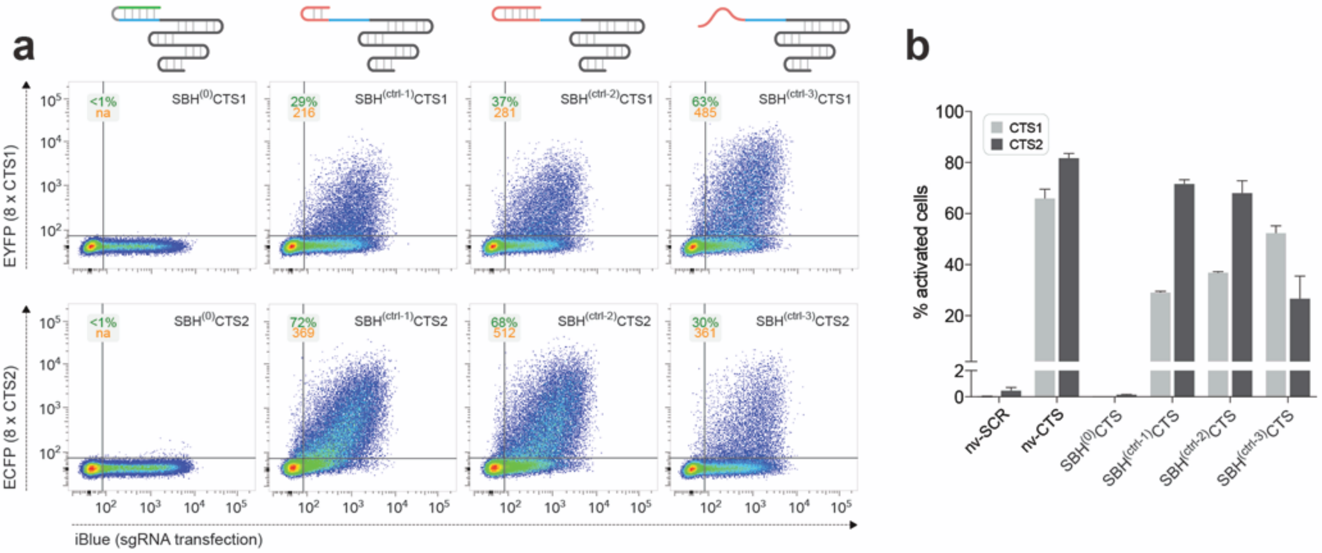


**Supplementary Figure 2. Control SBH-sgRNA mimics. (a)** HEK-293T cells were co-transfected with plasmids containing dCas9-VP64, 8xCTS1-mCMVp-EYFP or 8xCTS2-mCMVp-ECFP reporter, and the following SBH-sgRNAs: i) SBH$^{(0)}$CTS1 and SBH$^{(0)}$CTS2 (silent prototype SBH-sgRNAs with full spacer coverage); ii) SBH$^{(ctrl-1)}$CTS1 and SBH$^{(ctrl-1)}$CTS2 (control SBH-sgRNAs with accessible spacer and offset 5' end 10 bp hairpin structure); iii) SBH$^{(ctrl-2)}$CTS1 and SBH$^{(ctrl-2)}$CTS2 (control SBH-sgRNAs with accessible spacer and offset 5' end 20 bp hairpin structures); SBH$^{(ctrl-3)}$CTS1 and SBH$^{(ctrl-3)}$CTS2 (control SBH-sgRNAs with a scramble back-fold segment). Representative flow cytometry scatter plots (48h post-transfection) show reporter activation (EYFP, ECFP channels) plotted against sgRNA transfection (iBlue channel) for CTS1 and CTS2 spacers. Plots for SBH$^{(0)}$CTS1 and SBH$^{(ctrl-1)}$CTS1 are shared with Fig. 1d. Insets show % of activated cells (double iBlue$^{+ve}$ and EXFP$^{+ve}$, green) and median reporter fluorescence intensity for this population (orange). **(b)** Graph represents percentage of activated (EXFP$^{+ve}$) sgRNA expressing (iBlue$^{+ve}$) cells for all conditions in (a) and Fig. 1d (n=3, mean +/- s.d.).

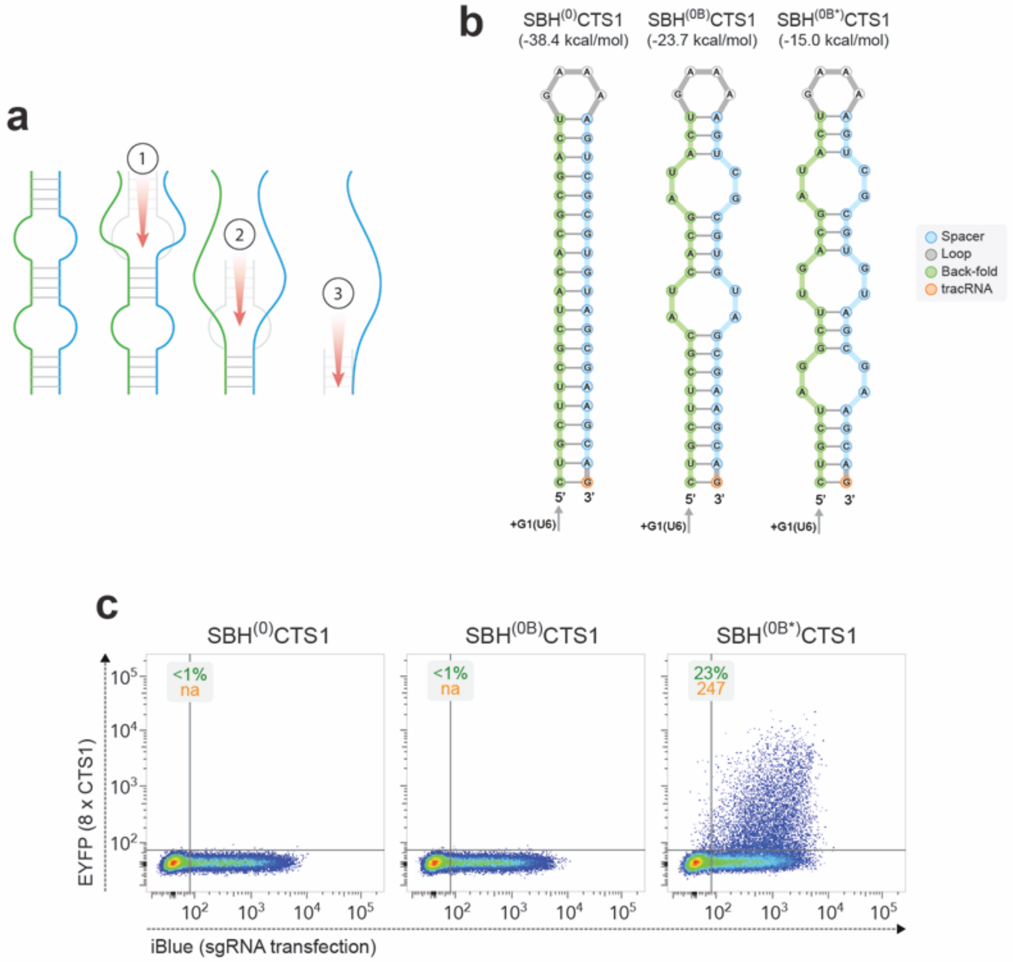


**Supplementary Figure 3. Design of SBHs with reduced thermodynamic stability. (a)** Hypothetical framework showing the low energy stepwise strand separation expected post-cleavage as a consequence of bulge incorporation into the SBH stem architecture. **(b)** Sequence and RNA secondary structure corresponding to the prototype SBH$^{(0)}$CTS1 design, and alternative architectures containing two (SBH$^{(0B)}$CTS1) and three (SBH$^{(0B*)}$CTS1) stem bulges. The calculated minimal free energy of each structure is shown in brackets. **(c)** Representative flow cytometry scatter plots (EYFP reporter fluorescence against iBlue sgRNA transfection) reveals complete CRISPR-TR silencing in both SBH$^{(0)}$CTS1 and SBH$^{(0B)}$CTS1 designs. In contrast, addition of a third bulge renders the sgRNA active, presumably due to excessive destabilisation of the stem structure. Insets show % of activated cells (double iBlue$^{+ve}$ and EYFP$^{+ve}$, green) and median reporter fluorescence intensity for this population (orange).

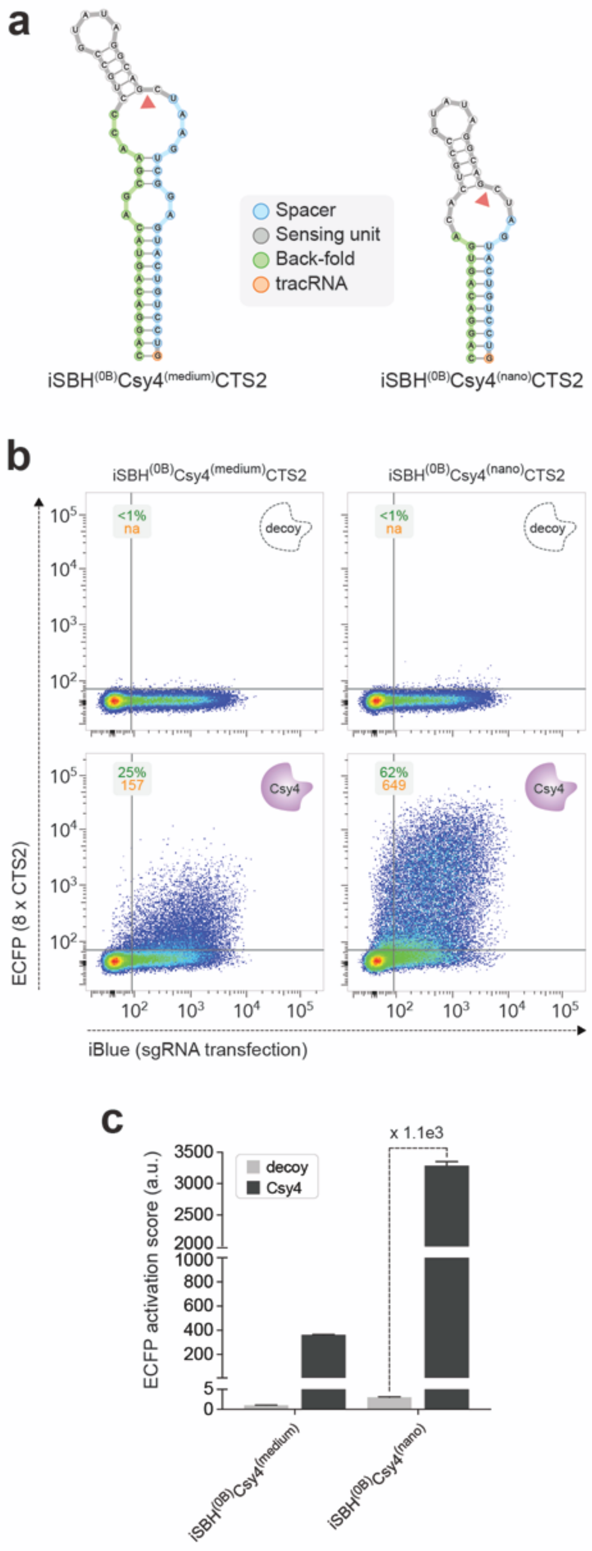


**Supplementary Figure 4. Optimisation of Csy4-responsive iSBH designs for CTS2. (a)** Sequence and RNA secondary structures of SBH$^{(0B)}$Csy4 medium, and nano stem design (red arrow = Csy4 cleavage site) for the CTS2 spacer. **(b)** Representative CRISPR-TR assay flow cytometry scatter plots for iSBH$^{(0B)}$Csy4$^{(medium)}$CTS2 and iSBH$^{(0B)}$Csy4$^{(nano)}$CTS2 sgRNAs in the absence (OFF-state, top) and presence (ON-state, bottom) of Csy4. Insets show % of activated cells (double iBlue$^{+ve}$ and ECFP$^{+ve}$, green) and median reporter fluorescence intensity for this population (orange).**(c)** Quantification of reporter activation using the two iSBH variants in the presence of a decoy plasmid or Csy4 inducer from three biological replicates (n = 3, mean +/- SD, a.u. arbitrary units).

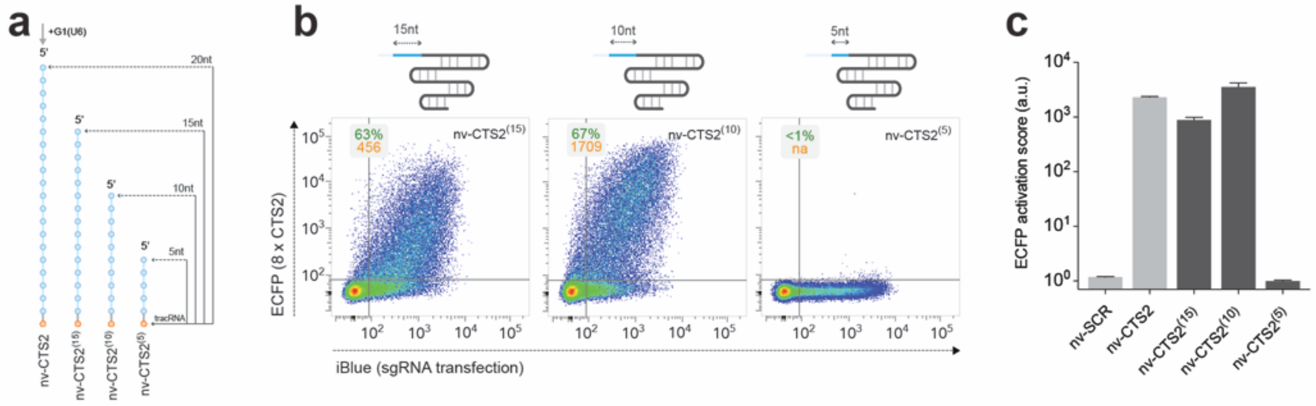


**Supplementary Figure 5. Effect of sgRNA spacer shortening on CRISPR-TR activity. (a)** Progressive shortening (5' to 3') of a native sgRNA spacer length (nv-CTS2 = native 20nt long spacer; nv-CTS2$^{(15, 10, 5)}$ = truncated spacers). **(b)** Representative flow cytometry scatter plots showing the impact of incremental spacer shortening (nv-CTS2$^{(15, 10, 5)}$) on reporter gene activation (axes represent ECFP reporter expression against sgRNA plasmid transfection for the three truncated sgRNA spacers). Insets show % of activated cells (double iBlue$^{+ve}$ and ECFP$^{+ve}$, green) and median reporter fluorescence intensity for this population (orange). **(c)** Quantification of ECFP reporter activation from matched experiments using the following sgRNAs: nv-SCR (control 20nt scramble spacer), nv-CTS2 (matching 20nt spacer), nv-CTS2$^{(15)}$(matching 15nt spacer), nv-CTS2$^{(10)}$(matching 10nt spacer), nv-CTS2$^{(5)}$(matching 5nt spacer) (n = 3, mean +/- s.d., a.u. arbitrary units).

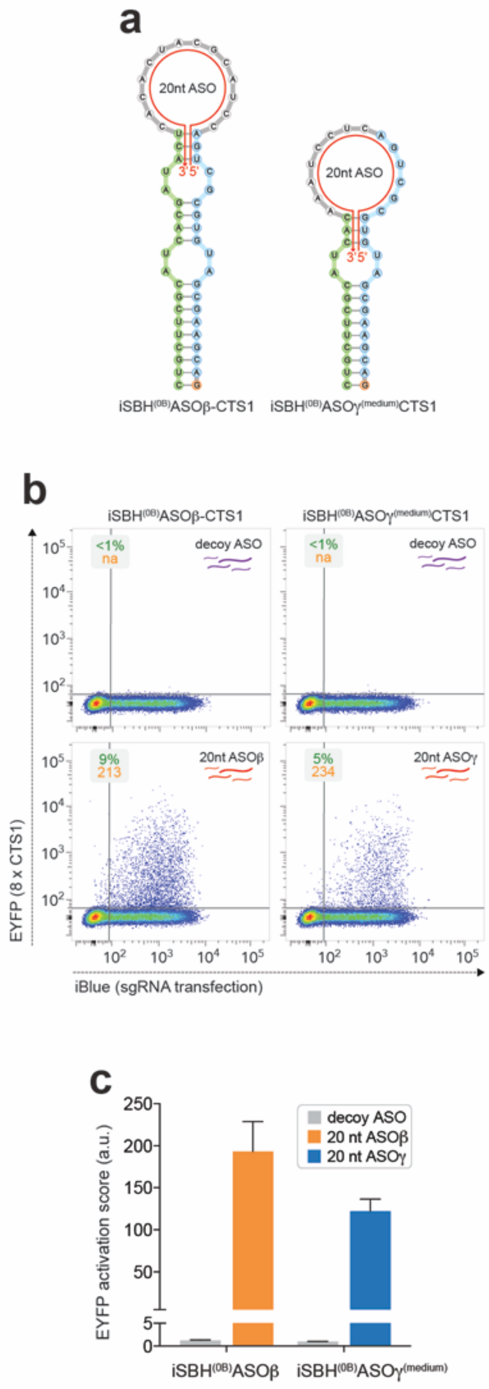


**Supplementary Figure 6. Evaluation of two ASO responsive iSBH designs. (a)** Sequence and RNA secondary structure of ASO-iSBH constructs with different stem length architectures. The iSBH$^{(0B)}$ASO β-CTS1 design was generated by replacing the 4nt loop in SBH$^{(0B)}$CTS1 with a 14nt sensing loop complementary to ASOβ. Extrapolating from the observation that shorter protein-responsive iSBH stems tend to display superior behavior, we also engineered iSBH$^{(0B)}$ASOγ$^{(medium)}$CTS1 by converting the distal bulge into a 14nt ASO sensing-loop, which incorporates 6 spacer nucleotides and was evolved to enforce an open ssRNA secondary structure. **(b)** Representative flow cytometry scatter plots (EYFP reporter fluorescence against iBlue sgRNA transfection) using the two ASO-responsive iSBH sgRNAs designs, in the presence of a decoy ASO (OFF-state) or active cognate ASO (ON-state). Insets show % of activated cells (double iBlue$^{+ve}$ and EYFP$^{+ve}$, green) and median reporter fluorescence intensity for this population (orange). **(c)** Quantification of reporter activation scores for all conditions shown in (b) (n = 3 biological replicates, mean +/- s.d., a.u. arbitrary unit).

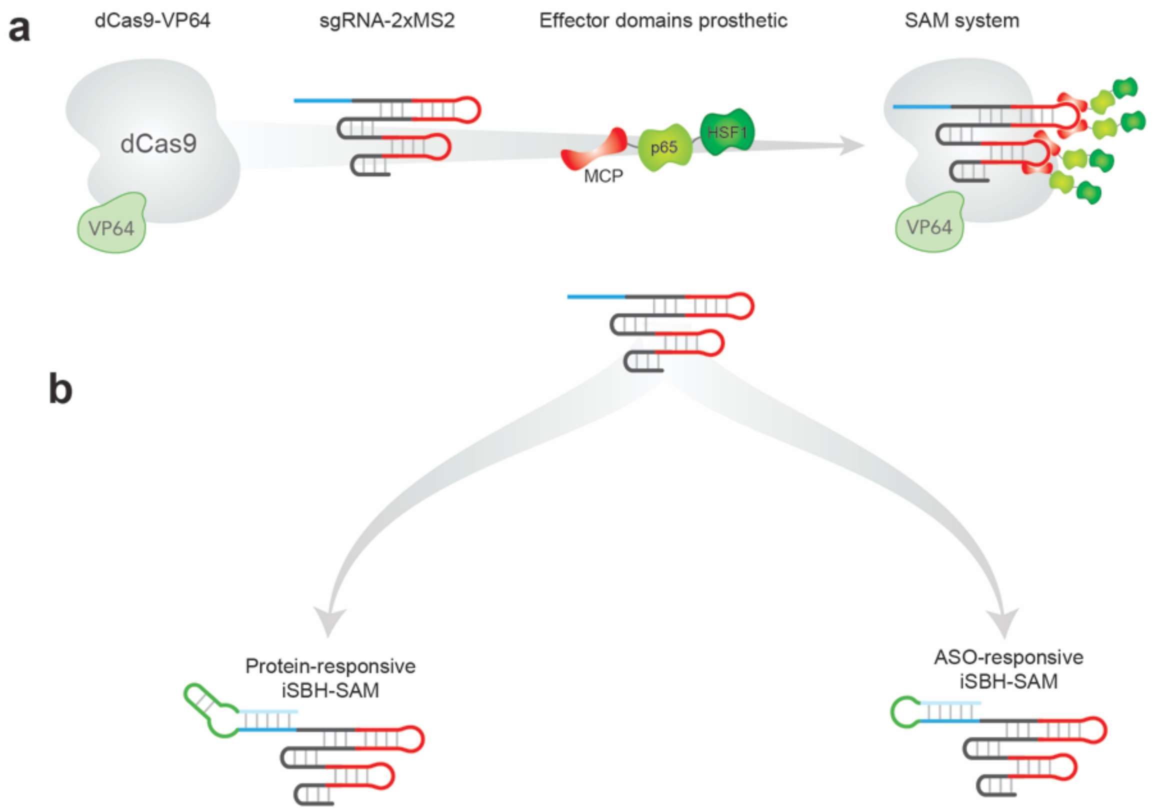


**Supplementary Figure 7. iSBH for SAM-based CRISPR-TR. (a)** Schematic diagram detailing the SAM system for single sgRNA CRISPR-mediated transcriptional activation of endogenous target genes[11]. dCas9-VP4 is complexed with a modified sgRNA-2xMS2 (red hairpins) capable of recruiting additional 4x p65-HSF1 effectors through MS2-MCP interactions. **(b)** Modification of sgRNA-2xMS2 with protein or ASO-responsive iSBH.

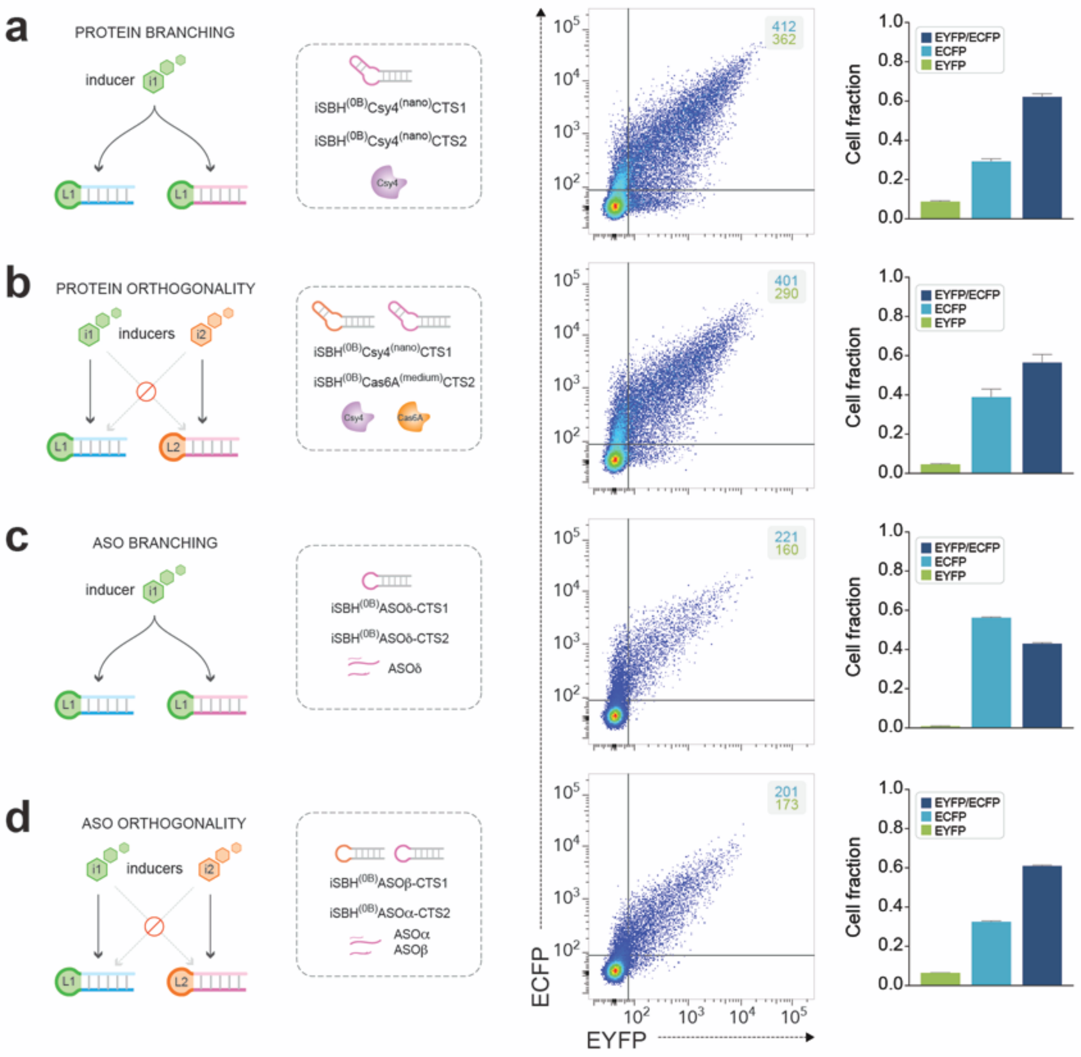


**Supplementary Figure 8. Evaluation of reporter co-activation in single cells. (a-d)** Flow cytometry analysis (48h post-transfection) of cell fractions expressing EYFP and ECFP reporter targets in four gene network module implementations: **(a)** branched activation with single protein inducer (related to Fig. 4b); **(b)** simultaneous orthogonal activation with two protein inducers (related to Fig. 4d); **(c)** branched activation with single ASO inducer (related to Fig. 5b); **(d)** ASO-mediated orthogonal activation of two reporter genes (related to Fig. 5d). Panels show the schematic of each gene module, system components, raw flow cytometry scatter plots and quantification of EYFP$^{+ve}$, ECFP$^{+ve}$, and double positive subpopulation frequencies within activated cells (n = 3 biological replicates, mean +/- s.d.). In all conditions between 40-60% of activated cells co-express both reporter genes. Insets show median reporter fluorescence intensity on the ECFP (light blue) and EYFP (green) channels in the double positive cell population (ECFP$^{+ve}$ and EYFP$^{+ve}$).

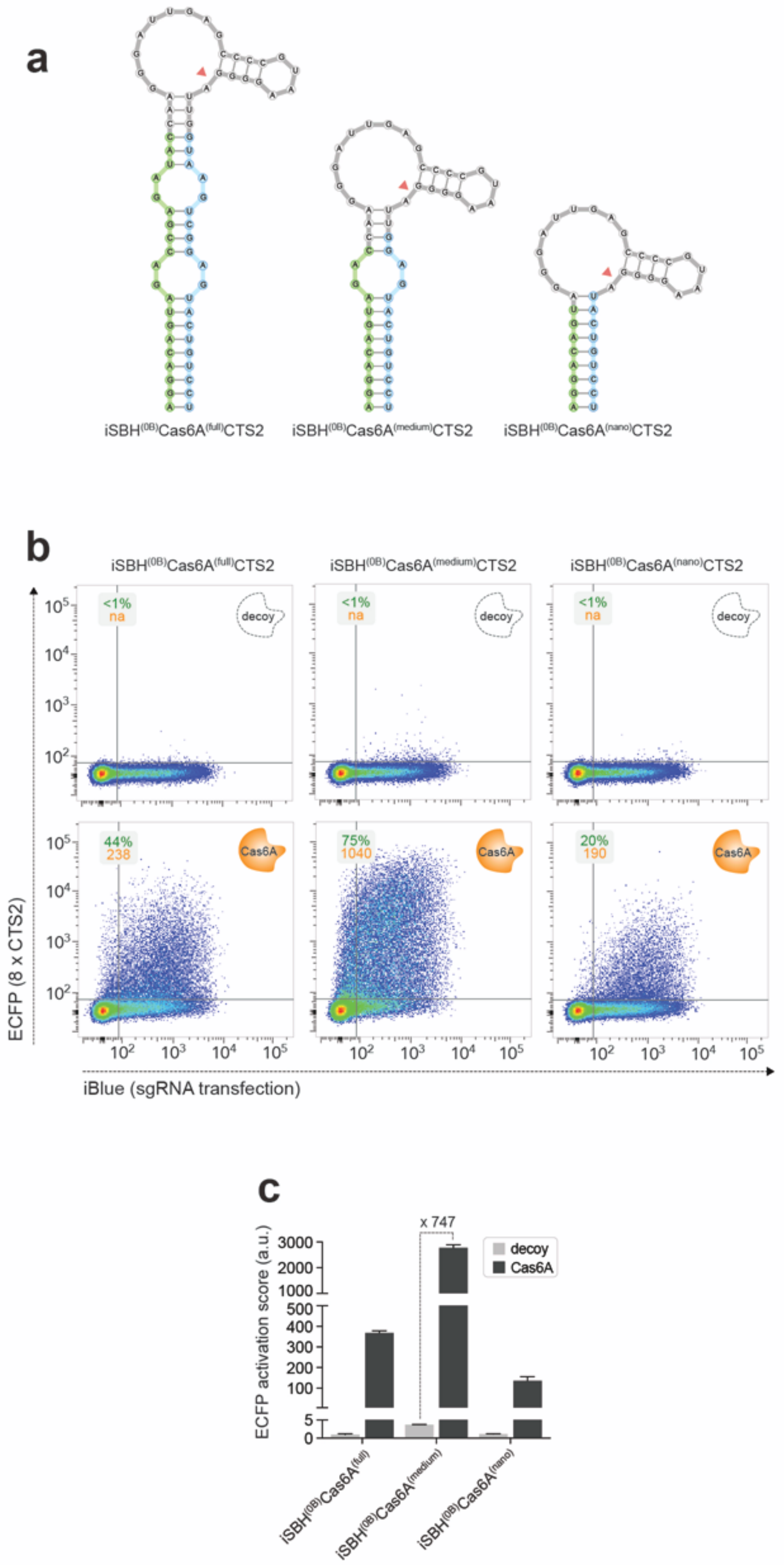


**Supplementary Figure 9. Optimisation of Cas6A-responsive iSBH designs for CTS2 spacer. (a)** Sequence and RNA secondary structures of SBH$^{(0B)}$Cas6A$^{(full)}$ sgRNA compared to the *medium*, and *nano* stems (CTS2 spacer; red arrow = Cas6A cleavage site) obtained by grafting the Cas6A RNA box onto the distal or proximal SBH$^{(0B)}$CTS2 bulge. **(b)** Representative CRISPR-TR assay flow cytometry scatter plots for iSBH$^{(0B)}$Cas6A$^{(full)}$CTS2, iSBH$^{(0B)}$Cas6A$^{(medium)}$CTS2 and iSBH$^{(0B)}$Cas6A$^{(nano)}$CTS2 sgRNAs in the absence (OFF-state, top) and presence (ON-state, bottom) of Cas6A. Insets show % of activated cells (double iBlue$^{+ve}$ and ECFP$^{+ve}$, green) and median reporter fluorescence intensity for this population (orange). **(c)** Quantification of ECFP reporter activation score using the three iSBH variants in the presence of a decoy plasmid or Cas6A inducer from three biological replicates (n = 3, mean +/- s.d., a.u. arbitrary units).

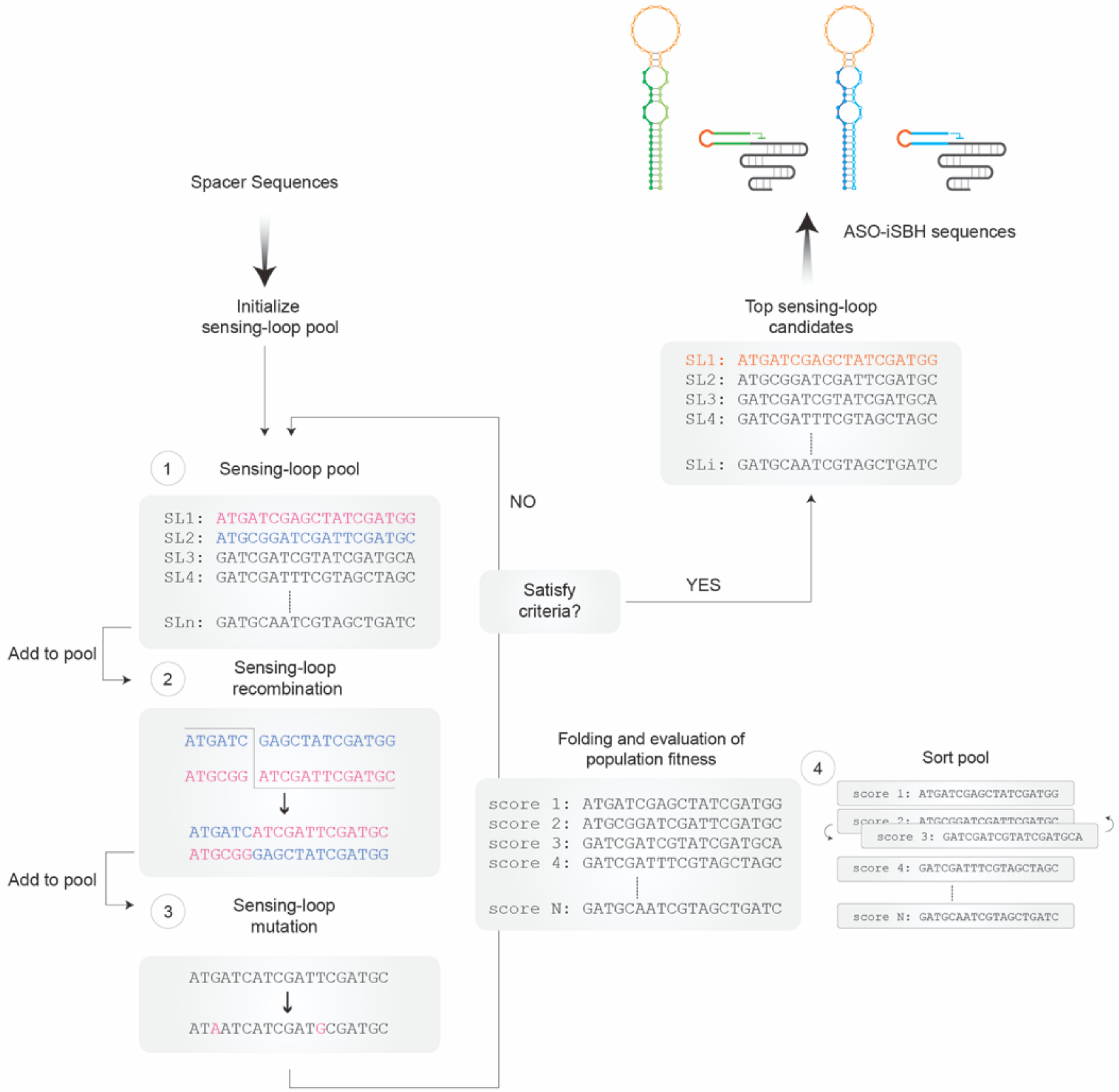


**Supplementary Figure 10. *In silico* evolution of a common ASO sensing-loop satisfying ASO-iSBH folding conditions across multiple spacers using *iSBHfold*.** For an input set of spacers (sp1, sp2, etc.) the algorithm aims to evolve a shared sensing-loop which can be grafted onto all SBH$^{(0B)}$spX while ensuring accessibility to ASO binding by enforcing structural constraints (open ssRNA loop conformation). Loops satisfying these conditions are evolved from an initial pool of 20nt RNA sequences [1]. A custom-made genetic algorithm is then run iteratively on this pool over several generations to optimise the sensing-loop sequences. For each iteration the following steps are performed: i) initial sequences are recombined to generate offspring sequences which are added to the original parent population [2]; ii) the pool is further enriched with sequences obtained by randomly mutating the existing segments as well as new fully randomised ones [3]. iii) finally, each sequence is folded within the corresponding iSBH using NUPACK[21] and attributed a folding score (FSi) measuring the similarity between the predicted RNA secondary structure and that of the target ASO-iSBH [4]. The ensuing pool is then sorted and only the fittest sequences are considered for the next iteration (repeat steps [1] to [4]). Once a user-defined criteria based on score or number of iterations is reached, the system outputs a list of top sensing-loop candidates used to create the ASO-responsive iSBHs.

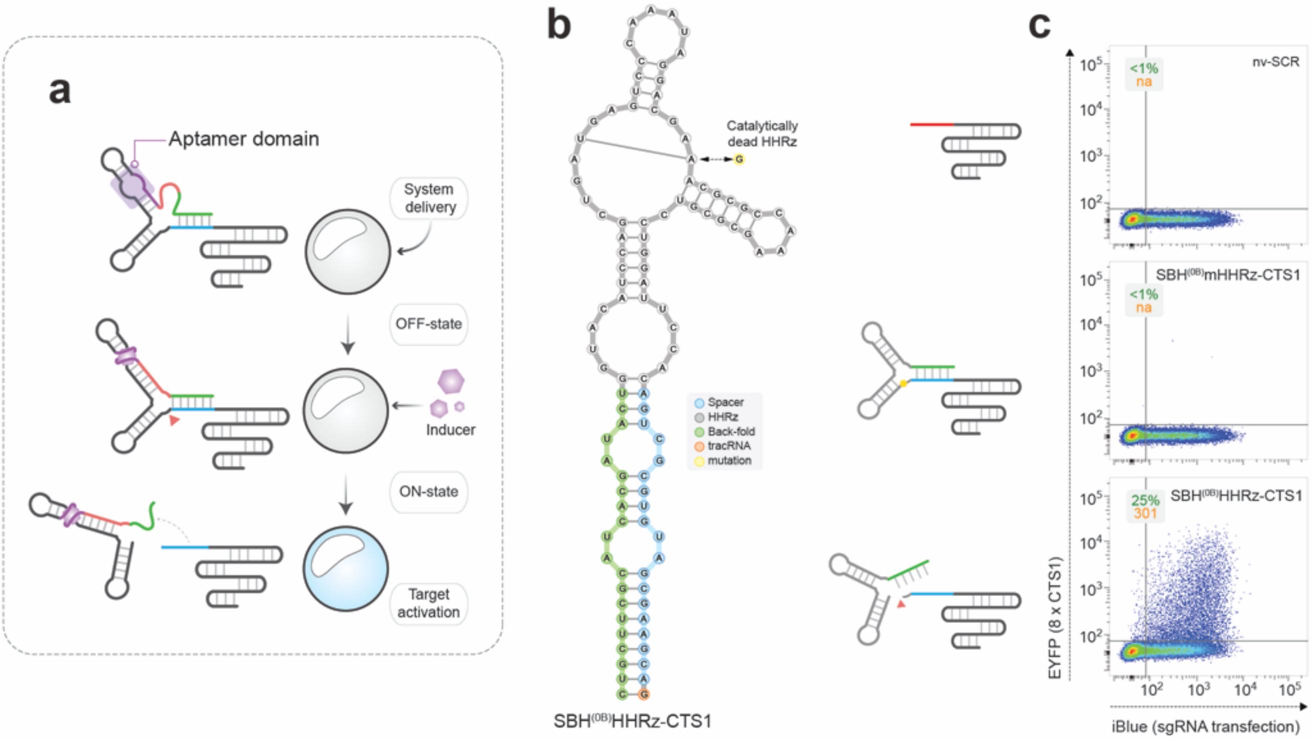


**Supplementary Figure 11. HHRz-based iSBH spacer release mechanism. (a)** Theoretical framework underlying the use of allosteric hammerhead ribozymes (aHHRz) for iSBH-sgRNA-based CRISPR-TR conditional control of gene expression. An iSBH equipped with an engineered aHHRz containing a ligand-sensing aptamer domain is delivered to cells. In the absence of a cognate ligand (OFF-state) the aHHRz assumes an inactive conformation, sequestering the spacer and inhibiting CRISPR-TR. Delivery of the cognate ligand promotes a change in conformation (strand displacement), which allows the aHHRZ to resume its catalytic activity. aHHRz cleavage facilitates back-fold dissociation from the spacer, rendering the sgRNA competent for CRISPR-TR-mediated activation of its programmed target gene (ON-state). **(b)** Sequence and RNA secondary structure of a prototype HHRz-SBH. The active HHRz is directly fused to the SBH$^{(0B)}$CTS1. A to G point mutation (yellow) renders the HHRz inactive (mHHRz). **(c)** Representative CRISPR-TR assay flow cytometry scatter plots with nv-SCR, SBH$^{(0B)}$HHRz-CTS1, and SBH$^{(0B)}$mHHRz-CTS1 sgRNAs (EYFP reporter fluorescence against iBlue sgRNA transfection). Insets show % of activated cells (double iBlue$^{+ve}$ and EYFP$^{+ve}$, green) and median reporter fluorescence intensity for this population (orange).

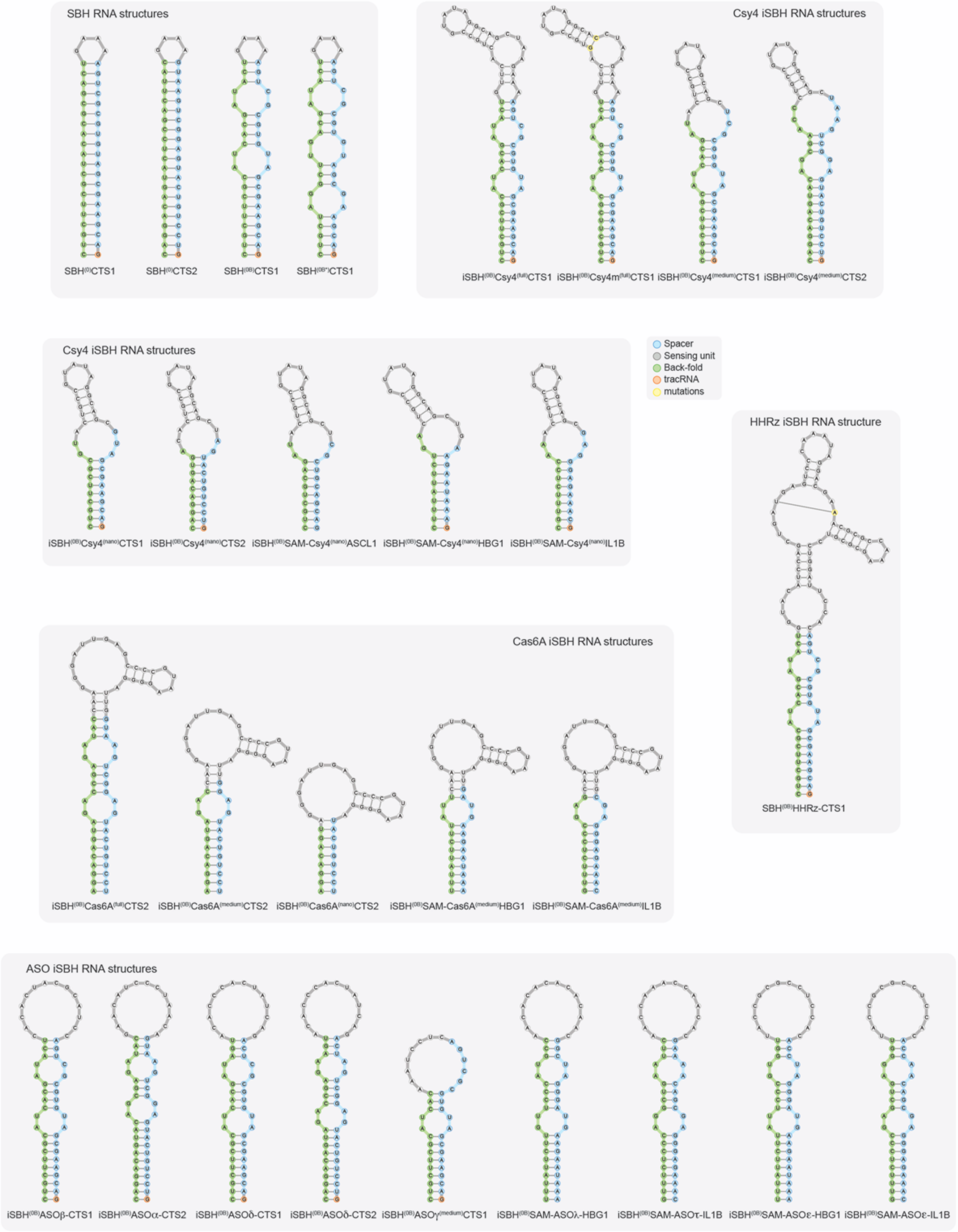


**Supplementary Figure 12.** Catalogue of silent (SBH) and and inducible (iSBH) designs used in the study organised by inducer and target type.

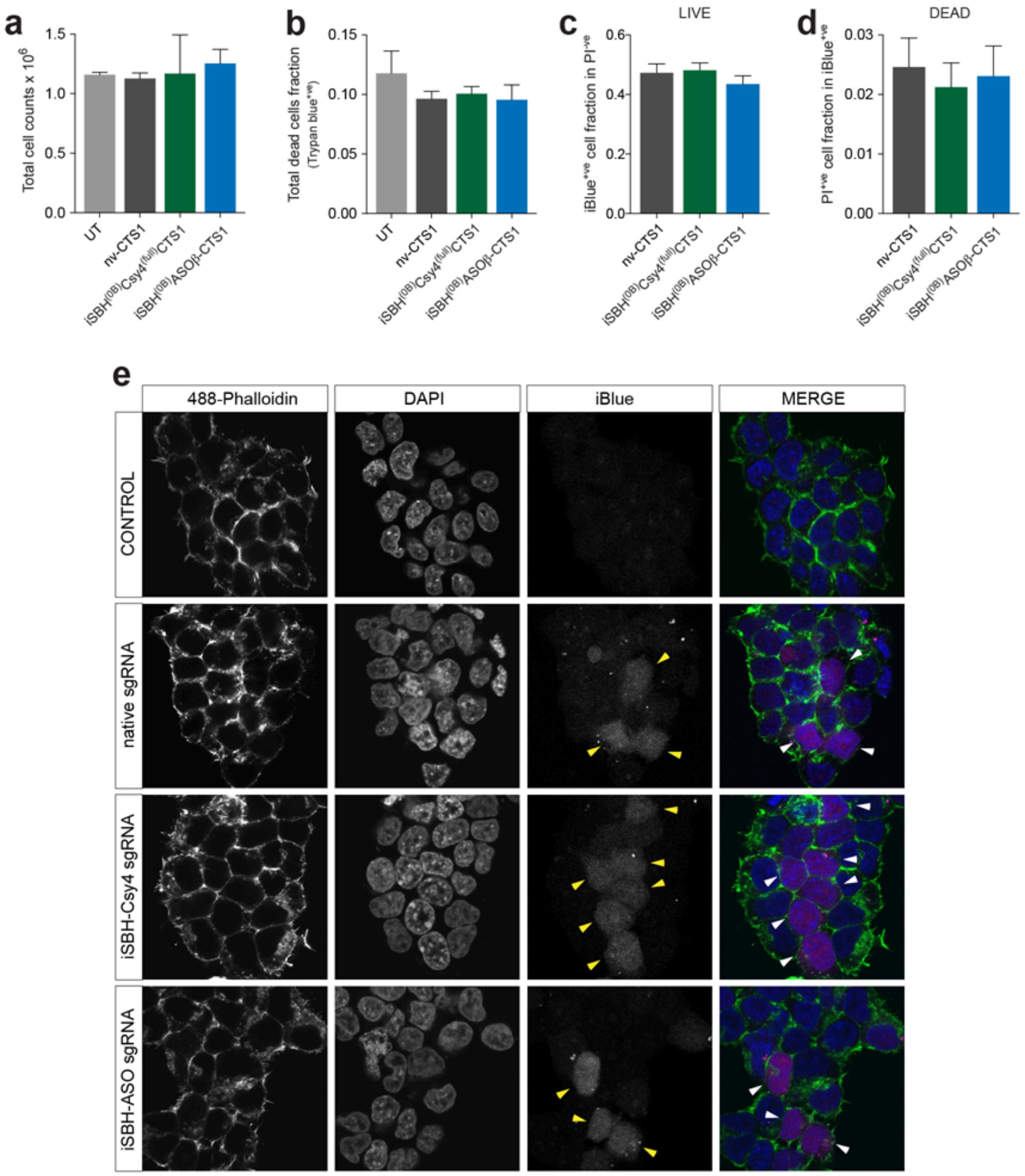


**Supplementary Figure 13. Analysis of cellular toxicity and stress following delivery of sgRNA variants.** HEK293-T cells were transfected with either a native sgRNA (nv-CTS1; NV), Csy4-responsive iSBH-sgRNA (iSBH$^{(0B)}$Csy4$^{(nano)}$CTS1; Csy4-SBH), or ASO-responsive iSBH-sgRNA (iSBH$^{(0B)}$ASO β-CTS1; ASO-SBH). Untransfected control cells were treated with PEI in the absence of plasmid DNA (UT). Cells were harvested 48h post-transfection, counted (hemocytometer) and stained with Propidium iodide (PI) before flow cytometry analysis. **(a)** Total cell counts for UT, NV, Csy4-SBH and ASO-SBH conditions. **(b)** Fraction of total dead cells measured by Trypan Blue staining. **(c)** Flow cytometry quantification of sgRNA expressing cell fraction (iBlue+ve) amongst live cells (PI-ve).**(d)** Flow cytometry quantification of dead cell fraction (PI+ve) amongst cells expressing sgRNAs (iBlue+ve). **(e)** Confocal microscopy analysis of cellular morphology (488-Phalloidin, green) and nuclear integrity (DAPI, blue) in UT cells compared to cells transfected with nv-CTS1, iSBH$^{(0B)}$Csy4$^{(nano)}$CTS1 and iSBH$^{(0B)}$ASOβ-CTS1 sgRNA variants (iBlue, magenta).

**U6-sgRNA-6xT cassette** (U6 promoter, scramble spacer segment with 2x BbsI sites, sgRNA scaffold, terminator)

GAGGGCCTATTTCCCATGATTCCTTCATATTTGCATATACGATACAAGGCTGTTAGAGAGATAATTGGAATT
AATTTGACTGTAAACACAAAGATATTAGTACAAAATACGTGACGTAGAAAGTAATAATTTCTTGGGTAGTTT
GCAGTTTTAAAATTATGTTTTAAAATGGACTATCATATGCTTACCGTAACTTGAAAGTATTTCGATTTCTTG
GCTTTATATATCTTGTGGAAAGGACGAAACACCGGGTCTTCGAGAAGACCTGTTTTAGAGCTAGAAATAGCA
AGTTAAAATAAGGCTAGTCCGTTATCAACTTGAAAAAGTGGCACCGAGTCGGTGCTTTTTT

**Human codon optimized Cas6A** (Kozak sequence, 5' NLS, TTCas6A)

GCCACCATGGCCCCAAAGAAGAAGCGGAAGGTCGGTATCCACGGAGTCCCAGCAGCCGAAAACCTG
TACTTCCAATCCAATGCAGCTAGCGTGCTTGCCGCGCTTGTGCTCGTGTTGGAAGGGGAAGGCCTC
CCGGAGCCGTTGGGTCTTAGGGGTTTCTTTTACGGCCTTTTGCGCGAGGTAGCGCCAGAGGTACAC
GATCAAGGAGAAAATCCTTTTGCTCTGGGATTCGGCGGGAGGGAAGGCGCAGCTTGGGCTAGGGTT
AGTCTTCTCGTAGAAGGGTTGTATGCGCGACTCGCACCTAGACTCTATGCGCTTGAAGGTGAGGAA
GTTCGCCTTGGACCTCCGTTCCGGGTACGCGCAGTCTTGCAAGAGGGCCATCCTTGGGCTGGAGTT
TCAACGTATCCACGCTTGTTCCAGGGACCTCCCTCCAGAGATCTGGCCCTCCGATTTGCAAGTCCA
ACCTTCTTCCGCCGGAAAGGCGTTCACTACCCCGTGCCGGAACCTCGCCTGGTTCTGGAGTCCCTC
CTGCGGCGGTTGGAAGCATTCGGTCCACTTAAAGCCCCGGAAGGAGTGAGAGAAGCTCTTCTGGAG
AGAACCACGGTGCGATCATTGGAGGGACGCACACTTCCGGCGAGGACGGAAGTTGACACAGCTGGA
TTTGTAGGCAGGGTCGTGTACCACCTGCCTAGAGCGACAGAGGAAGAAGCACTCTGGTTGTCTGCG
TTGGGCCGATTCGCGTTCTATTCAGGAGTAGGTGCAAAGACCAGCTTGGGATATGGTAGAGCACGA
GCAGAATCTGCGTAG

**Supplementary Figure 14.** DNA sequences of U6-sgRNA-6xT cassette and the human codon optimized Cas6A.

| Oligo/Primer name | Sequence (5'-3') |
|---|---|
| Fwd_sgRNA-cassette_SpeI | TAGACTAGTGAGGGCCTATTTCCCATG |
| Rev_sgRNA-cassette_BcoDI | CTAGTCTCTGCTAGCAAAAAAGCACCGACTCGG |
| Fwd_SV40_NcoI | CTAGCCTAACTCCGCCCAGTTCC |
| Rev_SV40 | GATCAGATCCGAAAATGGATATAC |
| Fwd_iBlue | GTATATCCATTTTCGGATCTGATCGCCACCATGTCGGTACC |
| Rev_iBlue_BsrGI | CTACTATGTACATCATTTGGACTGAGACTGTGC |
| HHRz_oligo_1 | TAGGAAGACTACACCGCTGCTTCGCTACACGCGACTGGTACA |
| HHRz_oligo_2 | CGCGTTTCGTCCTATTTGGGACTCATCAGCTGGATGTACCAGTCGCGTGTAG |
| HHRz_oligo_2m | CGCGTCTCGTCCTATTTGGGACTCATCAGCTGGATGTACCAGTCGCGTGTAG |
| HHRz_oligo_3 | CCAAATAGGACGAAACGCGCCAAAGCGCGTCCTGGATTCCACAGTCGCGTG |
| HHRz_oligo_3m | CCAAATAGGACGAGACGCGCCAAAGCGCGTCCTGGATTCCACAGTCGCGTG |
| HHRz_oligo_4 | CTAGAAGACTAAAACTGCTTCGCTACACGCGACTGTGGAATCC |
| HHRz_oligo_flanking _fwd | TAGGAAGACTACACCGCTGC |
| HHRz_oligo_flanking _rev | CTAGAAGACTAAAACTGCTTCGCT |
| Fwd_pX330_NLS | GACCTGCATCCCAAGCTTACGCGTGCCACCATGGCCCCAAAGAAGAAGCG |
| Rev_pX330_NLS | GGTCGCTAGCTGCATTGGATTGGAAGTACAGGTTTTCGGCTGCTGGGACTCCGTG |

**Supplementary Table 1.** Cloning oligonucleotides and PCR primers used in the study.

| NAME | Plasmid backbone | Insert (5' overhang CACC, 3' overhang GTTT) |
|---|---|---|
| nv-SCR | pcDNA3.1_U6-sgRNA-6T_SV40-iBlue-pA | GGGTCTTCGAGAAGACCT |
| nv-CTS1 | pcDNA3.1_U6-sgRNA-6T_SV40-iBlue-pA | GAGTCGCGTGTAGCGAAGCA |
| nv-CTS2 | pcDNA3.1_U6-sgRNA-6T_SV40-iBlue-pA | GTAAGTCGGAGTACTGTCCT |
| SBH(0)CTS1 | pcDNA3.1_U6-sgRNA-6T_SV40-iBlue-pA | GCTGCTTCGCTACACGCGACTGAAAAGTCGCGTGTAGCGAAGCA |
| SBH(0)CTS2 | pcDNA3.1_U6-sgRNA-6T_SV40-iBlue-pA | GCAGGACAGTACTCCGACTTACGAAAGTAAGTCGGAGTACTGTCCT |
| SBH(ctrl-1)CTS1 | pcDNA3.1_U6-sgRNA-6T_SV40-iBlue-pA | GAAAGCAGCCGAAAGGCTGCTTTCAGTCGCGTGTAGCGAAGCA |
| SBH(ctrl-1)CTS2 | pcDNA3.1_U6-sgRNA-6T_SV40-iBlue-pA | GACAAGCAGCCGAAAGGCTGCTTGTCGTAAGTCGGAGTACTGTCCT |
| SBH(ctrl-2)CTS1 | pcDNA3.1_U6-sgRNA-6T_SV40-iBlue-pA | GCAGGACAGTACTCCGACTTACGAAAGTAAGTCGGAGTACTGTCCTGAGTCGCGTGTAGCGAAGCA |
| SBH(ctrl-2)CTS2 | pcDNA3.1_U6-sgRNA-6T_SV40-iBlue-pA | GCTGCTTCGCTACACGCGACTGAAAAGTCGCGTGTAGCGAAGCAGGTAAGTCGGAGTACTGTCCT |
| SBH(ctrl-3)CTS1 | pcDNA3.1_U6-sgRNA-6T_SV40-iBlue-pA | GTAGCGATCAGTAGTCAGAATGAAAAGTCGCGTGTAGCGAAGCA |
| SBH(ctrl-3)CTS2 | pcDNA3.1_U6-sgRNA-6T_SV40-iBlue-pA | GACGTAGAGTTAGTTAGCTAGAGAAAGTAAGTCGGAGTACTGTCCT |
| SBH(5)CTS1 | pcDNA3.1_U6-sgRNA-6T_SV40-iBlue-pA | GCGCTACACGCGACTGAAAAGTCGCGTGTAGCGAAGCA |
| SBH(5)CTS2 | pcDNA3.1_U6-sgRNA-6T_SV40-iBlue-pA | GAGTACTCCGACTTACGAAAGTAAGTCGGAGTACTGTCCT |
| SBH(10)CTS1 | pcDNA3.1_U6-sgRNA-6T_SV40-iBlue-pA | GCACGCGACTGAAAAGTCGCGTGTAGCGAAGCA |
| SBH(10)CTS2 | pcDNA3.1_U6-sgRNA-6T_SV40-iBlue-pA | GTCCGACTTACGAAAGTAAGTCGGAGTACTGTCCT |
| SBH(15)CTS1 | pcDNA3.1_U6-sgRNA-6T_SV40-iBlue-pA | GGACTGAAAAGTCGCGTGTAGCGAAGCA |
| SBH(15)CTS2 | pcDNA3.1_U6-sgRNA-6T_SV40-iBlue-pA | GCTTACGAAAGTAAGTCGGAGTACTGTCCT |
| wt-CTS1(5) | pcDNA3.1_U6-sgRNA-6T_SV40-iBlue-pA | GAAGCA |
| wt-CTS2(5) | pcDNA3.1_U6-sgRNA-6T_SV40-iBlue-pA | GTCCT |
| wt-CTS1(10) | pcDNA3.1_U6-sgRNA-6T_SV40-iBlue-pA | GTAGCGAAGCA |
| wt-CTS2(10) | pcDNA3.1_U6-sgRNA-6T_SV40-iBlue-pA | GTACTGTCCT |
| wt-CTS1(15) | pcDNA3.1_U6-sgRNA-6T_SV40-iBlue-pA | GCGTGTAGCGAAGCA |
| wt-CTS2(15) | pcDNA3.1_U6-sgRNA-6T_SV40-iBlue-pA | GTCGGAGTACTGTCCT |
| SBH(0B)CTS1 | pcDNA3.1_U6-sgRNA-6T_SV40-iBlue-pA | GCTGCTTCGCATCACGATACTGAAAAGTCGCGTGTAGCGAAGCA |
| SBH(03xB)CTS1 | pcDNA3.1_U6-sgRNA-6T_SV40-iBlue-pA | GCTGCTAGGCTTGACGATACTGAAAAGTCGCGTGTAGCGAAGCA |
| iSBH(0B)Csy4(full)CTS1 | pcDNA3.1_U6-sgRNA-6T_SV40-iBlue-pA | GCTGCTTCGCATCACGATACTGTTCACTGCCGTATAGGCAGCTAAGAAAAGTCGCGTGTAGCGAAGCA |
| iSBH(0B)Csy4m(full)CTS1 | pcDNA3.1_U6-sgRNA-6T_SV40-iBlue-pA | GCTGCTTCGCATCACGATACTGTTCAGTGCCGTATAGGCACCTAAGAAAAGTCGCGTGTAGCGAAGCA |
| iSBH(0B)Csy4(medium)CTS | pcDNA3.1_U6-sgRNA-6T_SV40-iBlue-pA | GCTGCTTCGCATCACGATACTGCCGTATAGGCAGCTCGCGTGTAGCGAAGCA |
| iSBH(0B)Csy4(medium)CTS | pcDNA3.1_U6-sgRNA-6T_SV40-iBlue-pA | GCAGGACAGTACAGCGAACCCTGCCGTATAGGCAGCTAAGTCGGAGTACTGTCCT |
| iSBH(0B)Csy4(nano)CTS1 | pcDNA3.1_U6-sgRNA-6T_SV40-iBlue-pA | GCTGCTTCGCGTACTGCCGTATAGGCAGCGTAGCGAAGCA |
| iSBH(0B)Csy4(nano)CTS2 | pcDNA3.1_U6-sgRNA-6T_SV40-iBlue-pA | GCAGGACAGTGACACTGCCGTATAGGCAGCTAGTACTGTCCT |
| SAM_nv-SCR | SAM_U6-sgRNA-2xMS2 | GGGTCTTCGAGAAGACCT |
| SAM_nv-HBG1 | SAM_U6-sgRNA-2xMS2 | GGCTAGGGATGAAGAATAAA |
| SAM_nv-IL1B | SAM_U6-sgRNA-2xMS2 | GAAAACAGCGAGGGAGAAAC |
| iSBH(0B)Csy4(nano)ASCL1 | SAM_U6-sgRNA-2xMS2 | GCTGCTGCAGATACTGCCGTATAGGCAGCTCGCTGCAGCAG |
| iSBH(0B)Csy4(nano)HBG1 | SAM_U6-sgRNA-2xMS2 | GCTTTATTCTGACTGCCGTATAGGCAGCTGAAGAATAAA |
| iSBH(0B)Csy4(nano)IL1B | SAM_U6-sgRNA-2xMS2 | GCGTTTCTCCAAACTGCCGTATAGGCAGCGAGGGAGAAAC |
| iSBH(0B)Cas6A(full)CTS2 | pcDNA3.1_U6-sgRNA-6T_SV40-iBlue-pA | GAGGACAGTAGACCGAGATACCAAGGGATTGAGCCCCGTAAGGGGATTGGTAAGTCGGAGTACTGTCCT |
| iSBH(0B)Cas6A(medium)CT | pcDNA3.1_U6-sgRNA-6T_SV40-iBlue-pA | GAGGACAGTAGACCAAGGGATTGAGCCCCGTAAGGGGATTGGAGTACTGTCCT |
| iSBH(0B)Cas6A(nano)CTS2 | pcDNA3.1_U6-sgRNA-6T_SV40-iBlue-pA | GAGGACAGTAGGGATTGAGCCCCGTAAGGGGATACTGTCCT |
| iSBH(0B)Cas6A(medium)HB | SAM_U6-sgRNA-2xMS2 | GTTTATTCTTATTCAAGGGATTGAGCCCCGTAAGGGGATTGATGAAGAATAAA |
| iSBH(0B)Cas6A(medium)IL1 | SAM_U6-sgRNA-2xMS2 | GTTTCTCCCGAGCAAGGGATTGAGCCCCGTAAGGGGATTGCGAGGGAGAAAC |
| iSBH(0B)ASO1-CTS1 | pcDNA3.1_U6-sgRNA-6T_SV40-iBlue-pA | GCTGCTTCGCATCACGATACTCACACTACGCATCCAGTCGCGTGTAGCGAAGCA |
| iSBH(0B)ASO2-CTS2 | pcDNA3.1_U6-sgRNA-6T_SV40-iBlue-pA | GCAGGACAGTACAGCGAGATACGAACATCCCTAACAGTAAGTCGGAGTACTGTCCT |
| iSBH(0B)ASO-CTS1 | pcDNA3.1_U6-sgRNA-6T_SV40-iBlue-pA | GCTGCTTCGCATCACGATAGTACACCCACTATCAGACTCGCGTGTAGCGAAGCA |
| iSBH(0B)ASO-CTS2 | pcDNA3.1_U6-sgRNA-6T_SV40-iBlue-pA | GCAGGACAGTAGACCGAGAAGTACACCCACTATCAGACTAGTCGGAGTACTGTCCT |
| iSBH(0B)ASO1(medium)CTS | pcDNA3.1_U6-sgRNA-6T_SV40-iBlue-pA | GCTGCTTCGCATCACAAATCCTCAGTCGCGTGTAGCGAAGCA |
| iSBH(0B)ASO1-HBG1 | SAM_U6-sgRNA-2xMS2 | GTTTATTCTTGTTCCCATGCCAACACACACACAACGGCTAGGGATGAAGAATAAA |
| iSBH(0B)ASO2-IL1B | SAM_U6-sgRNA-2xMS2 | GGTTTCTCCCAGGCTGAATTCAACCAAACCAACACGAAAACAGCGAGGGAGAAAC |
| iSBH(0B)ASO-HBG1 | SAM_U6-sgRNA-2xMS2 | GTTTATTCTTATTCCCGTGGTTACCGCGCCTCCACACCTAGGGATGAAGAATAAA |
| iSBH(0B)ASO-IL1B | SAM_U6-sgRNA-2xMS2 | GGTTTCTCCCGAGCTGAGGGTTACCGCGCCTCCACACCAACAGCGAGGGAGAAAC |
| SBH(0B)HHRz-CTS1 | SAM_U6-sgRNA-2xMS2 | GCTGCTTCGCATCACGATACTGGTACATCCAGCTGATGAGTCCCAAATAGGACGAAACGCGCCAAAGCGCGTCCTGGATTCCACAGTCGCGTGTAGCGAAGCA |
| SBH(0B)mHHRz-CTS1 | SAM_U6-sgRNA-2xMS2 | GCTGCTTCGCATCACGATACTGGTACATCCAGCTGATGAGTCCCAAATAGGACGAGACGCGCCAAAGCGCGTCCTGGATTCCACAGTCGCGTGTAGCGAAGCA |

**Supplementary Table 2.** Sequence of native sgRNA spacer and SBH / iSBH hairpins used in the study together with the corresponding cloning vectors.

| NAME | Sequence | Corresponding ASO-iSBH |
|---|---|---|
| ASOα-14 | T*G*T*TAGGGATGT*T*C*G | iSBH$^{(0B)}$ASOα-CTS2 |
| ASOα-255 | T*A*C*T*GTTAGGGATGTTCGTAT*C*T*C*G | iSBH$^{(0B)}$ASOα-CTS2 |
| ASOβ | A*C*T*G*GATGCGTAGTGT*G*A*G*T | iSBH$^{(0B)}$ASOβ-CTS1 |
| ASOγ | C*A*C*G*CGACTGAGGATT*T*G*T*G | iSBH$^{(0B)}$ASOγ$^{(medium)}$CTS1 |
| ASOα | T*A*C*T*GTTAGGGATGTT*C*G*T*A | iSBH$^{(0B)}$ASOα-CTS2 |
| ASOδ | A*G*T*C*TGATAGTGGGTG*T*A*C*T | iSBH$^{(0B)}$ASOδ-CTS1 |
| | | iSBH$^{(0B)}$ASOδ-CTS2 |
| ASOλ | G*C*C*G*TTGTGTGTGTGT*T*G*G*C | iSBH$^{(0B)}$ASOλ-HBG1 |
| ASOτ | T*T*C*G*TGTTGGTTTGGT*T*G*A*A | iSBH$^{(0B)}$ASOτ-IL1B |
| ASOε | G*G*T*G*TGGAGGCGCGGT*A*A*C*C | iSBH$^{(0B)}$ASOε-HBG1 |
| | | iSBH$^{(0B)}$ASOε-IL1B |

**Supplementary Table 3.** ASO sequences used in the study.

| Target mRNA | Forward Primer Sequence (5'-3') | Reverse Primer Sequence (5'-3') |
|---|---|---|
| GAPDH | AACAGCGACACCCACTCCTC | CATACCAGGAAATGAGCTTGACAA |
| dCas9-VP64 (SAM) | AACCTATGCCCACCTGTTCG | ATCCAGGATTGTCTTGCCGG |
| HBG1 | GTTGTCTACCCATGGACCCA | TCTCCCAAGGAAGTCAGCAC |
| IL1B | CGAATCTCCGACCACCACTA | AGGGAAAGAAGGTGCTCAGG |

**Supplementary Table 4.** RT-qPCR primers used in the study.
