# Supplementary material for: Rational design of inducible CRISPR guide RNAs for de novo assembly of transcriptional programs
Source: Nat Commun. 2017 Mar 3;8:14633. doi: 10.1038/ncomms14633 (PMC5339017; doi:10.1038/ncomms14633)
Supplement: Supplementary Software — The SBHFold algorithm has been created to facilitate the design of ASO responsive iSBH structures sharing a common sensing loop sequence (see Supplementary Fig. 10 for in-depth explanation of the algorithm). Additionally, several smaller programs were included in the SBHFold suite to allow users to carry out the following operations: (1) Generate all SBH(x)-spacer hairpins for a given spacer sequence, where x varies between 0 and 20 (or length of the spacer sequence) and stands for the number of free spacer nucleotides (RNA sequences, minimal free energy (MFE), predicted structure, and cloning primers are outputted for each entry); (2) Given a SBH(0)- spacer hairpin, create all bulged SBHs by sliding a user defined bulged pattern along the SBH stem (RNA sequences, minimal free energy (MFE), predicted structure, and cloning primers are outputted for each entry); (3) Evolve a portion of the SBH RNA sequence to satisfy a user-defined RNA secondary structure, by searching over all possible sequences; (4) Evolve a portion of the SBH RNA sequence to satisfy a user-defined RNA secondary structure using a custom made genetic algorithm (optimization from RNA sequence pool). The entire suite has been written in Java on the Eclipse IDE (Luna Service Release 2 (4.4.2)) and a running commented example for each of the aforementioned programs is provided in the ‘Main.java' file. A more comprehensive step-by-step example illustrating the use of SBHFold for the evolution of a shared ASO sensing loop is provided in the same file. After unzipping the archive, the SBHFold program can be imported in the Eclipse IDE (Luna Service Release 2 (4.4.2)) as a new project. Note that SBHFold requires installation of the command line version of NUPACK to run correctly. This source code can be downloaded for free at http://www.nupack.org/downloads. The SBHFold zip archive contains the following folders: 1) bin and src: contain the binary and source codes of the Java project respectively. Of import [file ncomms14633-s2.zip › SBHFold_v1/output/Chart.js-master/samples/pie.html]

Pie Chart
