# Supplementary material for: Rational design of inducible CRISPR guide RNAs for de novo assembly of transcriptional programs
Source: Nat Commun. 2017 Mar 3;8:14633. doi: 10.1038/ncomms14633 (PMC5339017; doi:10.1038/ncomms14633)
Supplement: Supplementary Software — The SBHFold algorithm has been created to facilitate the design of ASO responsive iSBH structures sharing a common sensing loop sequence (see Supplementary Fig. 10 for in-depth explanation of the algorithm). Additionally, several smaller programs were included in the SBHFold suite to allow users to carry out the following operations: (1) Generate all SBH(x)-spacer hairpins for a given spacer sequence, where x varies between 0 and 20 (or length of the spacer sequence) and stands for the number of free spacer nucleotides (RNA sequences, minimal free energy (MFE), predicted structure, and cloning primers are outputted for each entry); (2) Given a SBH(0)- spacer hairpin, create all bulged SBHs by sliding a user defined bulged pattern along the SBH stem (RNA sequences, minimal free energy (MFE), predicted structure, and cloning primers are outputted for each entry); (3) Evolve a portion of the SBH RNA sequence to satisfy a user-defined RNA secondary structure, by searching over all possible sequences; (4) Evolve a portion of the SBH RNA sequence to satisfy a user-defined RNA secondary structure using a custom made genetic algorithm (optimization from RNA sequence pool). The entire suite has been written in Java on the Eclipse IDE (Luna Service Release 2 (4.4.2)) and a running commented example for each of the aforementioned programs is provided in the ‘Main.java' file. A more comprehensive step-by-step example illustrating the use of SBHFold for the evolution of a shared ASO sensing loop is provided in the same file. After unzipping the archive, the SBHFold program can be imported in the Eclipse IDE (Luna Service Release 2 (4.4.2)) as a new project. Note that SBHFold requires installation of the command line version of NUPACK to run correctly. This source code can be downloaded for free at http://www.nupack.org/downloads. The SBHFold zip archive contains the following folders: 1) bin and src: contain the binary and source codes of the Java project respectively. Of import [file ncomms14633-s2.zip › SBHFold_v1/output/test.html]

AMS


BCube -- All Modulation Stems Module  
....................................  
2016/11/16 14:14:02
  
Input spacer : GTAAGTCGGAGTACTGTCCT
  
....................................  

#0, NAME: SB.0, TYPE: modulation
  
GAGGACAGTACTCCGACTTACGAAAGTAAGTCGGAGTACTGTCCTG
  
.((((((((((((((((((((....)))))))))))))))))))). | -37.2
  
FORWARD PRIMER: CACCGAGGACAGTACTCCGACTTACGAAAGTAAGTCGGAGTACTGTCCT  
REVERSE PRIMER: AAACAGGACAGTACTCCGACTTACTTTCGTAAGTCGGAGTACTGTCCTC

#1, NAME: SB.1, TYPE: modulation
  
GGGACAGTACTCCGACTTACGAAAGTAAGTCGGAGTACTGTCCTG
| U6G repeat
  
.(((((((((((((((((((....))))))))))))))))))).. | -35.6
  
FORWARD PRIMER: CACCGGGACAGTACTCCGACTTACGAAAGTAAGTCGGAGTACTGTCCT  
REVERSE PRIMER: AAACAGGACAGTACTCCGACTTACTTTCGTAAGTCGGAGTACTGTCCC

#2, NAME: SB.2, TYPE: modulation
  
GGACAGTACTCCGACTTACGAAAGTAAGTCGGAGTACTGTCCTG
| U6G repeat
  
(((((((((((((((((((....))))))))))))))))))).. | -35.6
  
FORWARD PRIMER: CACCGGACAGTACTCCGACTTACGAAAGTAAGTCGGAGTACTGTCCT  
REVERSE PRIMER: AAACAGGACAGTACTCCGACTTACTTTCGTAAGTCGGAGTACTGTCC

#3, NAME: SB.3, TYPE: modulation
  
GACAGTACTCCGACTTACGAAAGTAAGTCGGAGTACTGTCCTG
  
((((((((((((((((((....))))))))))))))))))... | -32.3
  
FORWARD PRIMER: CACCGACAGTACTCCGACTTACGAAAGTAAGTCGGAGTACTGTCCT  
REVERSE PRIMER: AAACAGGACAGTACTCCGACTTACTTTCGTAAGTCGGAGTACTGTC

#4, NAME: SB.4, TYPE: modulation
  
GCAGTACTCCGACTTACGAAAGTAAGTCGGAGTACTGTCCTG
  
.((((((((((((((((....))))))))))))))))..... | -27.9
  
FORWARD PRIMER: CACCGCAGTACTCCGACTTACGAAAGTAAGTCGGAGTACTGTCCT  
REVERSE PRIMER: AAACAGGACAGTACTCCGACTTACTTTCGTAAGTCGGAGTACTGC

#5, NAME: SB.5, TYPE: modulation
  
GAGTACTCCGACTTACGAAAGTAAGTCGGAGTACTGTCCTG
  
.(((((((((((((((....)))))))))))))))...... | -26.4
  
FORWARD PRIMER: CACCGAGTACTCCGACTTACGAAAGTAAGTCGGAGTACTGTCCT  
REVERSE PRIMER: AAACAGGACAGTACTCCGACTTACTTTCGTAAGTCGGAGTACTC

#6, NAME: SB.6, TYPE: modulation
  
GGTACTCCGACTTACGAAAGTAAGTCGGAGTACTGTCCTG
| U6G repeat
  
.((((((((((((((....))))))))))))))....... | -24.8
  
FORWARD PRIMER: CACCGGTACTCCGACTTACGAAAGTAAGTCGGAGTACTGTCCT  
REVERSE PRIMER: AAACAGGACAGTACTCCGACTTACTTTCGTAAGTCGGAGTACC

#7, NAME: SB.7, TYPE: modulation
  
GTACTCCGACTTACGAAAGTAAGTCGGAGTACTGTCCTG
  
((((((((((((((....))))))))))))))....... | -24.8
  
FORWARD PRIMER: CACCGTACTCCGACTTACGAAAGTAAGTCGGAGTACTGTCCT  
REVERSE PRIMER: AAACAGGACAGTACTCCGACTTACTTTCGTAAGTCGGAGTAC

#8, NAME: SB.8, TYPE: modulation
  
GACTCCGACTTACGAAAGTAAGTCGGAGTACTGTCCTG
  
.((((((((((((....))))))))))))......... | -21.5
  
FORWARD PRIMER: CACCGACTCCGACTTACGAAAGTAAGTCGGAGTACTGTCCT  
REVERSE PRIMER: AAACAGGACAGTACTCCGACTTACTTTCGTAAGTCGGAGTC

#9, NAME: SB.9, TYPE: modulation
  
GCTCCGACTTACGAAAGTAAGTCGGAGTACTGTCCTG
  
.(((((((((((....))))))))))).......... | -19.1
  
FORWARD PRIMER: CACCGCTCCGACTTACGAAAGTAAGTCGGAGTACTGTCCT  
REVERSE PRIMER: AAACAGGACAGTACTCCGACTTACTTTCGTAAGTCGGAGC

#10, NAME: SB.10, TYPE: modulation
  
GTCCGACTTACGAAAGTAAGTCGGAGTACTGTCCTG
  
.((((((((((....))))))))))........... | -17.6
  
FORWARD PRIMER: CACCGTCCGACTTACGAAAGTAAGTCGGAGTACTGTCCT  
REVERSE PRIMER: AAACAGGACAGTACTCCGACTTACTTTCGTAAGTCGGAC

#11, NAME: SB.11, TYPE: modulation
  
GCCGACTTACGAAAGTAAGTCGGAGTACTGTCCTG
  
.(((((((((....)))))))))............ | -15.6
  
FORWARD PRIMER: CACCGCCGACTTACGAAAGTAAGTCGGAGTACTGTCCT  
REVERSE PRIMER: AAACAGGACAGTACTCCGACTTACTTTCGTAAGTCGGC

#12, NAME: SB.12, TYPE: modulation
  
GCGACTTACGAAAGTAAGTCGGAGTACTGTCCTG
  
.((((((((....))))))))............. | -12.9
  
FORWARD PRIMER: CACCGCGACTTACGAAAGTAAGTCGGAGTACTGTCCT  
REVERSE PRIMER: AAACAGGACAGTACTCCGACTTACTTTCGTAAGTCGC

#13, NAME: SB.13, TYPE: modulation
  
GGACTTACGAAAGTAAGTCGGAGTACTGTCCTG
| U6G repeat
  
.(((((((....)))))))(((......))).. | -12.1
  
FORWARD PRIMER: CACCGGACTTACGAAAGTAAGTCGGAGTACTGTCCT  
REVERSE PRIMER: AAACAGGACAGTACTCCGACTTACTTTCGTAAGTCC

#14, NAME: SB.14, TYPE: modulation
  
GACTTACGAAAGTAAGTCGGAGTACTGTCCTG
  
(((((((....)))))))(((......))).. | -12.1
  
FORWARD PRIMER: CACCGACTTACGAAAGTAAGTCGGAGTACTGTCCT  
REVERSE PRIMER: AAACAGGACAGTACTCCGACTTACTTTCGTAAGTC

#15, NAME: SB.15, TYPE: modulation
  
GCTTACGAAAGTAAGTCGGAGTACTGTCCTG
  
.(((((....)))))..(((......))).. | -8.8
  
FORWARD PRIMER: CACCGCTTACGAAAGTAAGTCGGAGTACTGTCCT  
REVERSE PRIMER: AAACAGGACAGTACTCCGACTTACTTTCGTAAGC

#16, NAME: SB.16, TYPE: modulation
  
GTTACGAAAGTAAGTCGGAGTACTGTCCTG
  
.((((....))))...(((......))).. | -7.3
  
FORWARD PRIMER: CACCGTTACGAAAGTAAGTCGGAGTACTGTCCT  
REVERSE PRIMER: AAACAGGACAGTACTCCGACTTACTTTCGTAAC

#17, NAME: SB.17, TYPE: modulation
  
GTACGAAAGTAAGTCGGAGTACTGTCCTG
  
.(((....)))....(((......))).. | -6.4
  
FORWARD PRIMER: CACCGTACGAAAGTAAGTCGGAGTACTGTCCT  
REVERSE PRIMER: AAACAGGACAGTACTCCGACTTACTTTCGTAC

#18, NAME: SB.18, TYPE: modulation
  
GACGAAAGTAAGTCGGAGTACTGTCCTG
  
.((....)).....(((......))).. | -5.4
  
FORWARD PRIMER: CACCGACGAAAGTAAGTCGGAGTACTGTCCT  
REVERSE PRIMER: AAACAGGACAGTACTCCGACTTACTTTCGTC

#19, NAME: SB.19, TYPE: modulation
  
GCGAAAGTAAGTCGGAGTACTGTCCTG
  
..((.((((........)))).))... | -4.1
  
FORWARD PRIMER: CACCGCGAAAGTAAGTCGGAGTACTGTCCT  
REVERSE PRIMER: AAACAGGACAGTACTCCGACTTACTTTCGC
